# Adynamic and dynamic muscle transposition techniques for anal incontinence

**DOI:** 10.1093/gastro/gou014

**Published:** 2014-03-19

**Authors:** Goran Barišić, Zoran Krivokapić

**Affiliations:** Clinic for Digestive Surgery, First Surgical Clinic, Belgrade School of Medicine, University of Belgrade, Serbia

**Keywords:** anal incontinence, muscle transposition, gluteoplasty, graciloplasty, dynamization

## Abstract

Gracilis muscle transposition is well established in general surgery and has been the main muscle transposition technique for anal incontinence. Dynamization, through a schedule of continuous electrical stimulation, converts the fatigue-prone muscle fibres to a tonic fatigue-resistant morphology with acceptable results in those cases where there is limited sphincter muscle mass. The differences between gluteoplasty and graciloplasty, as well as the techniques and complications of both procedures, are outlined in this review. Overall, these techniques are rarely carried out in specialized units with experience, as there is a high revision and explantation rate.

## INTRODUCTION

Anal incontinence (AI) is a socially incapacitating condition which is distressing for patients and which severely impacts quality of life. It is estimated that AI affects up to 2% of the general population [1], although a systematic analysis by MacMillan *et al.* reported higher rates ranging between 11% and 15% [2]. The most common cause of AI is physical injury of the anal sphincter during childbirth, followed by iatrogenic injuries as a consequence of surgical procedures—including fistulotomy and haemorrhoidectomy—as well as direct trauma to the perineum after traffic accidents or war injuries. In most patients, conservative medical management is usually attempted as a first option; however in many cases, especially in traumatic AI, the treatment is surgical. The decision on which surgical procedure should be used depends upon the integrity of the anal sphincter, as it is still accepted practice that those with limited sphincter defects should undergo overlapping sphincteroplasty, whereas patients with intact sphincters should receive other treatment modalities, such as sacral nerve stimulation or biofeedback therapy. The problem exists in those patients who have absent or severely damaged, destroyed and unusable anal sphincters, where overlapping sphincteroplasty or other types of sphincter repair are not possible. In such cases, sphincter substitutions with an artificial bowel sphincter or with muscle transposition are the principal alternatives to the permanent stoma. This review focuses on muscle transposition techniques for the management of end-stage AI where other previous treatment efforts have failed.

Various skeletal muscles have in the past been used as neo-sphincters, in the hope that they will be able to maintain faecal continence. In most cases these were muscles in close proximity to the anus, which were harvested and wrapped around the anus in order to effectively replace the anal sphincters. The earliest reported attempt to apply this option used the *gluteus maximus* muscle at the beginning of the twentieth century (1902), when Charles Chetwood successfully performed transposition of the gluteus muscle to reinforce the sphincters and restore continence function in a patient who developed faecal incontinence after trauma [3]. Passive gracilis muscle transposition (without electrical stimulation) was first described by Pickrell *et al.* in 1952, when he attempted to restore continence function in four children [4]. Since the results of previous techniques were inconsistent and unpredictable, others were searching for additional solutions, using different skeletal muscles for the same purpose. Fedorov *et al.* [5] utilized the *adductor longus* whilst Hakelius *et al.* used free autogenous muscle transplants for the treatment of AI in children [6]. For this purpose, others have advocated smooth muscle transplants [7], where Hallan and colleagues employed an electrically stimulated sartorius neo-sphincter in a canine model [8]. The sartorius neo-sphincter showed excellent results when it was used in an animal model but, because the human sartorius muscle has segmental vascularisation, it has not proven to be clinically useful. The common and essential drawback of all passive muscle transposition techniques has been the inability of patients to voluntarily contract the transposed muscle and/or the inability of the muscle to sustain tonic contraction over prolonged periods of time. Although different techniques, using different muscles, have been proposed in the past for the treatment of AI, in practice only two muscles are still used for this purpose; the gluteus and the gracilis muscles.

## GLUTEOPLASTY (GLUTEUS MAXIMUS PLASTY)

**Figure 1.**
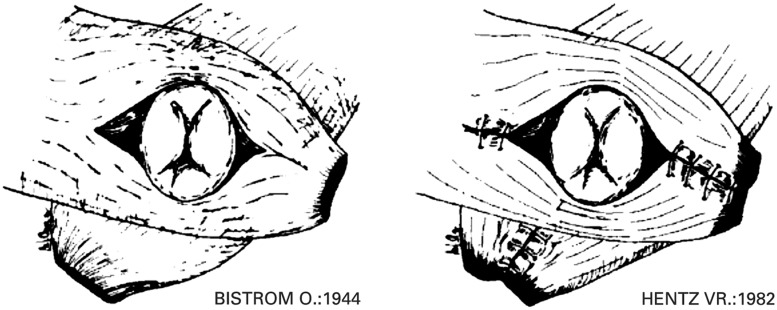
Alternative configurations for gluteus muscle transposition. (Reprinted with permission from Devesa JM, Vicente E, Enriquez JM *et al.* Total faecal incontinence – a new method of *gluteus maximus* transposition: preliminary results and report of previous experience with similar procedures. *Dis Colon Rectum* 1992;**35**:339–49).

Since Chetwood reported the first transposition of the *gluteus maximus* muscle in 1902, various modifications of the original technique have been described. In 1930, Chittenden used gluteal muscle flaps for anal reconstruction following an abdominoperineal resection [9], whilst Bistrom in 1944 transposed the gluteus muscle and pulled the rectal stump through a previously created aperture for the specific treatment of incontinence [10]. After World War II, this technique was almost abandoned until, in 1981, Bruining *et al.* reported a modified technique of gluteus muscle transposition in a 17-year-old female cyclist who had been run over by a lorry [11]. The operation was performed some 18 months after the injury, whereby the gluteus muscles were detached from the femur and wrapped around the anus in a scissor-like configuration. In 1982, Hentz described another technique, whereby the gluteus muscles were detached from their sacral and coccygeal origin and wrapped around the anus, the proposed mechanism of action being one in which the bilateral muscle slings encircling the rectum were thought to stabilize one another at a proper resting tension and fibre length [12]. In order to minimize donor site morbidity (namely, hip destabilization), Orgel and Kucan proposed the technique of a double-split *gluteus maximus* muscle flap, mobilizing only the inferior part of one muscle (usually the right side) at its insertion at the iliotibial band and femur [13]. These configurations are shown in Figure 1 [14].

A number of case reports or small series have been reported since 1980, using a variety of technical modifications based on proximally or distally-based muscle flaps [14–19]. In 1996, Guelinckx *et al.*, encouraged by reported results of dynamic cardiomyoplasty and dynamic graciloplasty, presented their results in seven patients treated by conventional gluteoplasty and four treated by dynamic gluteoplasty [20]. In theory, the use of the *gluteus maximus* muscle as a substitute for the anal sphincters has several advantages when compared with other muscles. It is a well vascularized, large and powerful voluntary muscle, supplied by the superior and inferior gluteal arteries, capable of forceful contraction. In this context, it is normally used as an auxiliary muscle to maintain faecal continence. It is innervated by the inferior gluteal nerve originating from the L5 and S1 nerve roots, which makes it functional even in cases of AI secondary to pudendal neuropathy or where there is denervation of the anal sphincters. Since it is an accessory muscle to continence, patients can be trained to use it quite easily. Due to the muscle morphology, active contraction and high tonus of the muscle can be maintained for longer periods than when the gracilis muscle is used, since prolonged contraction is of paramount importance for the maintenance of continence.

**Figure 2.**
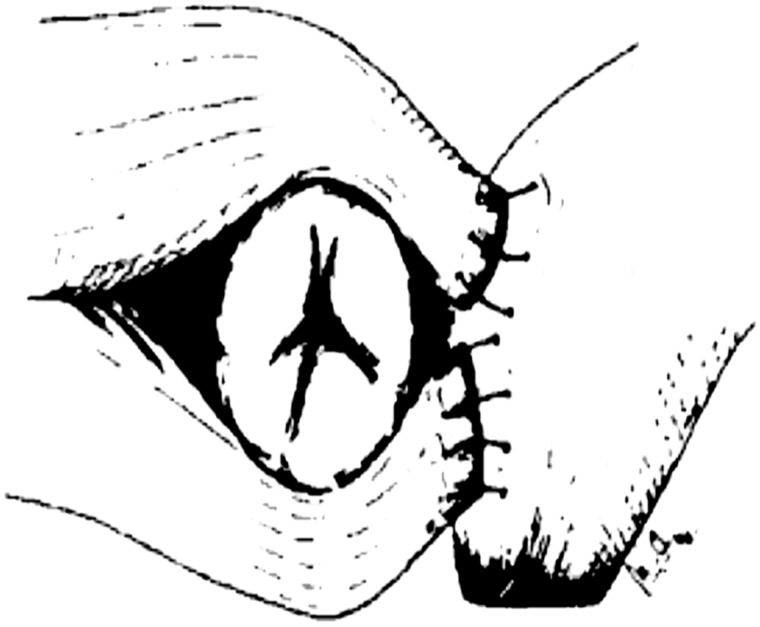
The Devesa technique of gluteoplasty. (Reprinted with permission from Devesa JM, Vicente E, Enriquez JM *et al.* Total faecal incontinence – a new method of *gluteus maximus* transposition: preliminary results and report of previous experience with similar procedures. *Dis Colon Rectum* 1992;**35**:339–49).

Therefore, deliberate closure of the anus is theoretically possible by its voluntary, forceful contraction following transposition. Furthermore, its neurovascular structures can easily be preserved during dissection and release from its attachments at the level of the sacrum, so the neurovascular pedicle after gluteoplasty has less traction as compared with the graciloplasty. Dissection is facilitated by following the direction of the muscle fibres and, in most cases, muscle wraps have sufficient length to reach the anal canal without excessive tension. When the dissection is complete, several kinds of wrap configurations are possible because the amount of the transferred muscle far exceeds the amount of muscle tissue in a normal anal sphincter, permitting complete encirclement. Further, the muscle redundancy is of value in those cases where there is post-operative atrophy; a not-uncommon occurrence after transposition. The best functional results have been obtained with a technique that uses the caudal parts of both gluteus muscles to encircle the anus, as shown in Figure 2 [14].

Unfortunately, despite these positive features, there are important drawbacks inherent in this technique. The *gluteus maximus* is a striated muscle and contains predominantly Type II muscle fibres, which make it a fatigue-prone muscle, incapable of sustaining continuous contraction for a prolonged period of time. Further, the muscle after dissection is bulky, making it technically demanding to tunnel and wrap around the anus, especially in patients with a deficient perineal body or where there is excessive scarring of the rectovaginal septum. There is also a problem with higher donor site morbidity, when compared with gracilis muscle transposition. In gluteoplasty, in most cases, the muscles from both sides should be harvested whereas, with a graciloplasty, only one muscle is usually taken. The *gluteus maximus* is a hip extensor, important for walking, running, standing up from a sitting position and for walking up stairs, so patients may experience some difficulties in everyday life during these specific activities. In most cases, sporting activity is dramatically reduced following this muscle transposition and, equally, this natural function of the muscle can make continence difficult to maintain when a patient is running or climbing stairs [21]. Finally, the surgical technique for gluteoplasty is more complex and challenging because the access to its neurovascular bundle is less familiar to the surgeon as compared with graciloplasty. These factors may well be some of the reasons why it has never gained the same level of popularity as the graciloplasty, even though the results of unstimulated gluteoplasty have been shown to be superior [22], or at least equal to those of an unstimulated graciloplasty [17].

### Indications and contraindications

As with other muscle transposition techniques, gluteoplasty is a last-choice option, designated for cases of end-stage incontinence when other treatment modalities are either not possible or have failed. The best candidates for this procedure are young patients with severe sphincter defects or destroyed sphincters not amenable to sphincteroplasty, or patients whose sphincteroplasties have failed. Gluteoplasty is also a good option for patients who may benefit from transposition of substantial muscle bulk, where there is an absence of excessive rectovaginal scarring. The main contra-indication for this procedure is injured or non-functional *gluteus maximus* muscles. Patients with AI secondary to spina bifida or a myelomeningocele should not be treated with a gluteoplasty because of impaired innervation. Equally, patients with Lériche syndrome are an absolute contra-indication for this procedure, since the vascular supply of the muscle is derived from the superior and inferior gluteal artery and is affected by internal iliac artery stenosis. In cases where the integrity and performance of the muscle are questionable, electromyography (EMG) should be performed, in order to rule out muscle dysfunction and denervation.

### Operative technique

Some surgeons use proximally-based muscle flaps, whereas others use distally-based muscle flaps. After pre-operative mechanical bowel preparation and antibiotic prophylaxis, the patient is operated upon in the prone jack-knife position. Bilateral oblique incisions are made lateral to the midline, extending to the ischial tuberosity. The inferior border of the *gluteus maximus* muscle is identified and the distal part of the muscle is detached from its sacro-coccygeal attachment, including the fascia attaching it to the sacrum. The muscle is then mobilized laterally, following the direction of its fibres, conserving the aponeurosis and taking care not to damage its neurovascular supply. The inferior gluteal artery and nerve are situated in the lower part of the muscle and exit the pelvis via the greater sciatic foramen. The mobilized part of the muscle is carefully divided, along the direction of its fibres, into two equal parts. After the same procedure is performed on the other side, two skin incisions are made a few centimetres (at least 2 cm) lateral to the anal verge and, by sharp and blunt dissection, subcutaneous tunnels are created around the anus, taking care not to injure the rectal or vaginal wall. Two separate tunnels are fashioned, in order to bring the ends of both harvested muscles to the peri-anal space. Following this manoeuvre, the previously divided muscle ends from both sides are passed around the anus, (two in front and two behind the anal canal), overlapped and sutured. A temporary diverting stoma is not mandatory, except in selected, high-risk cases.

### Outcomes

Complications following gluteoplasty are not uncommon. One review of the literature showed that the most frequent complication was wound infection, occurring in an average of 24% of patients [23]. Other, less frequent, complications include skin necrosis, anal canal necrosis, anal canal stricture, faecal impaction, obstructed defecation, chronic pain and donor site morbidity including posterior thigh numbness, dysaesthesia and severe chronic pain. Christiansen *et al.* reported wound infection in three out of seven patients after bilateral Gluteoplasty [17], with Hultman *et al.* reporting donor site morbidity and perirectal complications in 64% of patients in a retrospective analysis of 25 consecutive patients undergoing gluteoplasty for AI [19]. Puerta *et al.* reported a success rate of 70% using the gluteus transposition technique in 22 patients with AI [24]. Madoff *et al.* reported wound infection in 27% and anal stricture in 9% of a group of 11 patients treated with dynamic gluteoplasty [25].

In most reports, continence improved following Gluteoplasty; however, there are a limited number of studies with objective assessment of long-term results. Guelinckx *et al.* reported continence for stool in nine out of eleven patients, while seven patients were also continent for liquids [20]. Christiansen *et al.* found improvement in continence in three out of seven patients, while, in four, it remained unchanged [17]. Hultman *et al.* found that gluteoplasty was successful in restoring continence in 72% and partially successful in 16% of patients [19]. According to a review of the literature by Fleshner and Roberts, complete continence was achieved in 60% and partial continence in 36%, whilst total failure was observed in 4% of patients [23]. Combined data from 17 studies, encompassing 149 patients undergoing Gluteoplasty, showed a successful or partially successful outcome in 73%, with a complication rate of 38% overall. Since the first report by Chittenden, anorectal reconstruction following an abdomino-perineal resection (APR) using a supplementary gluteoplasty has received little attention, with only a few published reports [14, 24, 26].

### Dynamic gluteoplasty

Madoff *et al.* reported results from a prospective, multicentre study where electrostimulation of the transposed gluteus muscle was performed in order to improve results [25]. He initially achieved good outcomes in all patients, but a successful outcome was only maintained in five cases (45%) out of the 11 patients treated, where they concluded that this procedure should be considered experimental and used only in the context of a research study. Guelinckx *et al.* treated 11 patients, seven with conventional- and four with a dynamic Gluteoplasty, where all patients who had satisfactory results and were continent for both solid and liquid stool were in the dynamic gluteoplasty group [19]. By contrast, the electrostimulation threshold is much higher for a gluteoplasty than that required for a graciloplasty, effectively resulting in a shorter battery life when this technique is used.

## GRACILOPLASTY

More than 30 years after Pickrell *et al.* originally described this technique in 1952 [4], Corman *et al.* reported good results [27], with Faucheron *et al.* confirming that a significant proportion of patients may achieve satisfactory continence [28]. The gracilis is the most superficial adductor of the thigh and has little impact on motion of the lower extremity. Its natural function is to assist the legs in adduction and internal rotation so, when this muscle is used as a neo-sphincter, adjacent muscles take over its function. As a result, there is minimal donor site morbidity and patients can perform most of their everyday activities, including sports [29]. This superficial muscle has a constant and proximal neurovascular supply which enables effortless dissection and, in most cases, provides sufficient muscle length to overlap around the anus. Unfortunately, like all skeletal muscles, the gracilis has a preponderance of Type II, fatigue-prone, fast-twitch muscle fibres and is incapable of sustained and prolonged forceful contraction. At the same time, the natural function of this muscle has nothing in common with muscles responsible for continence, making it very difficult for a patient to learn how to contract and use the gracilis muscle as a true neo-sphincter. These patients could contract the anus only for a short period of time and only while they were consciously concentrating on its contraction.

Muscle fatigue and the inability of patients to voluntarily contract the transposed muscle were the main reasons why successful results of conventional graciloplasty were achieved in less than 50% of patients. Moreover, most of the patients regarded as successes had severe constipation due to outlet obstruction produced by over-tightening of the anus with the transposed muscle [30]. Tightening the muscle wrap around the anus reduces the power of contraction required for maintaining continence, but may create a problem in evacuation. In some cases, the gracilis has a long tendon with a relatively short muscle, and surgeons can over-tighten the anal canal during anal encirclement. Another possibility for improving the contractility of the muscle was to change its afferent nerve supply. It is well established that muscle fibre typing depends upon the afferent nerve where, in theory, anastomosing the pudendal nerve with the transposed gracilis may change the muscle properties. Although this is technically feasible, this procedure has resulted in an unacceptably high morbidity because the pudendal nerve is critical for normal bladder and pelvic floor function. Moreover, associated pudendal nerve damage in incontinent patients precludes this technique. The overall poor results obtained with conventional graciloplasty led to abandonment of the procedure.

### Operative technique

Most surgeons use the original surgical technique described by Pickrell *et al.* [4]. The operation is performed in the lithotomy position. Mechanical bowel cleansing is not mandatory, nor is a diverting stoma; however, antibiotic prophylaxis is necessary. The gracilis is the most superficial muscle and is exposed either by one large mid-thigh incision or by a few (1–3) separate incisions. The muscle is dissected from the adjacent tissue and small peripheral arteries (usually between one and four) are ligated. The distal tendon insertion at the tibial tuberosity is exposed by a separate small incision and the tendon is cut close to its insertion. The muscle is dissected proximally until the main neurovascular bundle is reached. In most cases, it is found approximately 8 cm from the proximal muscle insertion at the pubic bone. Lateral to the anus, two incisions are made, through which tunnels are created around the anus, usually by blunt digital dissection. The posterior tunnel should be wide enough to accommodate two fingers. This is usually not difficult to perform but the anterior tunnel can sometimes be difficult to dissect because the layer between the rectum and vagina is often very thin and, in many cases, extensively scarred. In this respect, great care should be taken in order to avoid inadvertent injury to either the rectum or the vagina. When dissection is extremely difficult, it may be wise to make an additional incision posterior to the vagina, in order to prevent rectal injury. The anterior tunnel should be wide enough to accommodate three fingers, especially if a gamma wrap configuration is to be created.

Next, a tunnel connecting the thigh and perineal incisions should be created by combined digital and sharp dissection of the strong *fascia lata*. This tunnel should be sufficiently wide to easily accommodate the gracilis muscle, in order to prevent muscle entrapment, ischaemia and necrosis. There are several wrap configurations which can be performed, depending upon the length of the gracilis muscle and the perineal wound. The aim is to encircle the anus with muscular tissue as much as possible. In cases where there is a long muscular part and the need for muscle bulk between the rectum and the vagina, a gamma loop is feasible. In cases with extensive scarring on the anterior side and insufficient space to accommodate the muscle, an epsilon loop can be created to transpose the muscle bulk posteriorly. In cases of a short muscular part, an alpha loop is preferred [31]. Once the position of the muscle is determined, the distal gracilis tendon is sutured to the periostium of the pubic bone at the contralateral side, using non-absorbable sutures in cases of gamma and epsilon configurations. Alternatively, the tendon may be sutured to the periostium of the ischial tuberosity or to the subcutaneous skin. In cases of an alpha loop, this is performed at the ipsilateral side. In all cases, care should be taken not to over-tighten the anal canal, which will result in outlet obstruction. Overall, as stated, reports of unstimulated graciloplasty have shown success in less than 50% of cases, although some have claimed that the continence rate was improved more than 80% [27, 30, 32, 33].

### Dynamic graciloplasty

The physiological basis for dynamic gracilopasty was developed from experimental studies in animals. Here it was found that low-frequency electrical stimulation of the muscle could change the normal muscle fibre pattern and transform fibre Type II, fatigue-prone muscles into fibre Type I, fatigue-resistant muscles [8, 34]. This process can change skeletal muscles like the gracilis into a muscle with properties of a sphincter muscle. With this technique, the main cause for failure of the passive graciloplasty—such as an inability to voluntarily contract muscle and to maintain prolonged contraction—can be overcome. The introduction of muscle electro-stimulation techniques were based upon research using the *latissimus dorsi* muscle to assist a failing heart in dynamic cardiomyoplasty [35], which renewed interest in gracilis muscle transposition. Although the first electrical stimulation was reported by Dickson in 1968 for the treatment of faecal incontinence after an operation for anorectal agenesis [36], it was Cor Baeten from Maastricht who, in 1986, implanted the first neurostimulator in a patient who had already undergone a conventional graciloplasty 10 years previously for anal atresia.

The development of this new technique, called dynamic graciloplasty, was modified by Baeten *et al.* and Williams *et al.* [37, 38], who independently developed the concept of transforming fast, fatigue-prone muscle fibres into slow, fatigue-resistant fibres by continuous electrical stimulation. In Italy, Cavina *et al.* used graciloplasty for anal sphincter reconstruction after an abdominoperineal excision [39]. In this study, electrical stimulation was performed for just a brief period after surgery, using an external device. In England, Williams and his group from the London Hospital directly and periodically stimulated the nerve trunk using an external device. In Maastricht, Baeten *et al.* used a permanent, implantable stimulator for direct muscular electrostimulation. In this respect, direct nerve stimulation is more physiological and requires a lower stimulation level, thus maximizing battery life where muscle electrodes are more secure and where there is less chance of lead displacement. This procedure became the most popular form of a muscle neo-sphincter because of its simplicity, the consistency of the neurovascular supply and because of minor donor site morbidity.

### Operative technique

The surgical technique of dynamic graciloplasty is similar to that of a conventional graciloplasty. The difference is that, before the gracilis is transposed to the perianal tunnel, electrodes are sutured to the muscle. Two electrodes, positive and negative, are inserted near the entry point of the obturator nerve into the muscle. The anode should be positioned first and distally to the nerve entrance. Intra-operative test stimulation enables the best positioning of the cathode. When the best contraction is achieved, the electrodes are anchored in place to the epimysium. Once the electrodes are anchored, a subcutaneous tunnel is created to the lower abdomen at the ispilateral side, where a pocket is fashioned in the subcutaneous tissue, designed to accommodate the electrical stimulator. Electrodes are connected to the stimulator and all wounds are closed. This part of the procedure may be performed at the same time as the graciloplasty (single stage procedure) or as a separate procedure approximately six weeks later (two-stage procedure). Matzel *et al.* found no difference in the complication rate between the single- and two-staged procedures [40]. To prevent seroma formation, the leg should be bandaged. A diverting stoma is not necessary, since it has been found that a stoma does not decrease the wound complication rate [25]. Six weeks after surgery, when the patient has recovered, the stimulation training protocol can commence. This protocol lasts eight weeks and consists of intermittent stimulation of the neo-sphincter, which gradually increases in duration and intensity [41].

### Outcomes

Dynamic graciloplasty is a major procedure and many complications have been reported. The most significant sources of morbidity were wound complications. In a prospective multi-institutional study involving 139 patients from 12 centres, Madoff *et al.* reported major wound complications in 32% of patients and minor wound complications in 29% [25]. Tendon detachment was recorded in 3%, pain in 22% and device/stimulation problems in 11% of patients. Necrosis of the neo-anus, as well as the gracilis muscle, was also reported. Moreover, sixty-six (48%) of the 138 reported complications in this study required one or more repeat operations. Functional failure, unrelated to any specific complication, was recorded in 40% of all the failure group. Constipation is also reported after graciloplasty in approximately 16% of patients and, in some cases, is due to over-tightening of the anus with the gracilis. Insufficient contraction of the gracilis muscle is a further complication caused by muscular or stimulation problems. As dynamic graciloplasty and its troubleshooting require considerable experience, it is a procedure which should only be performed in high-volume centres. In this respect, Madoff *et al.* reported significant differences in the complication rates and outcomes when highly experienced centres were compared with low-volume units, showing an incidence of major wound complication in 17.4 vs 33.1%, respectively [25].

There are many reports of efficacy with dynamic graciloplasty in the literature, with success rates ranging from 45 to 80% [42–47]. Most studies have reported small numbers of patients with an overall improvement of continence in about 50% of cases and with a follow-up ranging from 7 months to 4 years. Again, experienced centres did better than inexperienced ones, the reported overall success rate being 80% in experienced centres, compared with only 47% in inexperienced centres [25]. Heterogeneity in reported success rates may be related to the learning curve associated with this complex procedure. It may also be related to the length of the follow-up and different tools and definitions used to measure outcomes.

## CONCLUSIONS

The caveat to these procedures has been their high morbidity with a range of wound infections, high rates of re-operation, complications specific for implanted devices and the problem of evacuatory difficulty. The long-term consequences of chronic electrostimulation remain unknown since there are concerns that chronic electrostimulation may decrease muscle fibre diameter and lead to muscle degeneration and atrophy by affecting the collateral blood supply [48, 49]. Another important disadvantage is the high cost of the procedure. However, studies have shown that there is a considerable long-term cost benefit from graciloplasty, when compared with the costs of a colostomy [50].

In summary, muscle transposition techniques continue to have a limited role in the treatment of faecal incontinence, although the results are not always predictable. Graciloplasty appears superior to Gluteoplasty, as it is technically easier, has more neurovascular consistency and less donor site morbidity. Given the high revision and explantation rates with dynamic graciloplasty, it should only be considered when there is a deficient sphincter, precluding other techniques such as sacral and peripheral nerve stimulation and where a permanent colostomy is the only other viable management alternative.

**Conflict of interest**: none declared.
